# Supplementary figures and images for: A combination of genomics and transcriptomics provides insights into the distribution and differential mRNA expression of type VI secretion system in clinical Klebsiella pneumoniae
Source: mSphere. 2024 Mar 4;9(3):e00822-23. doi: 10.1128/msphere.00822-23 (PMC10964426; doi:10.1128/msphere.00822-23)

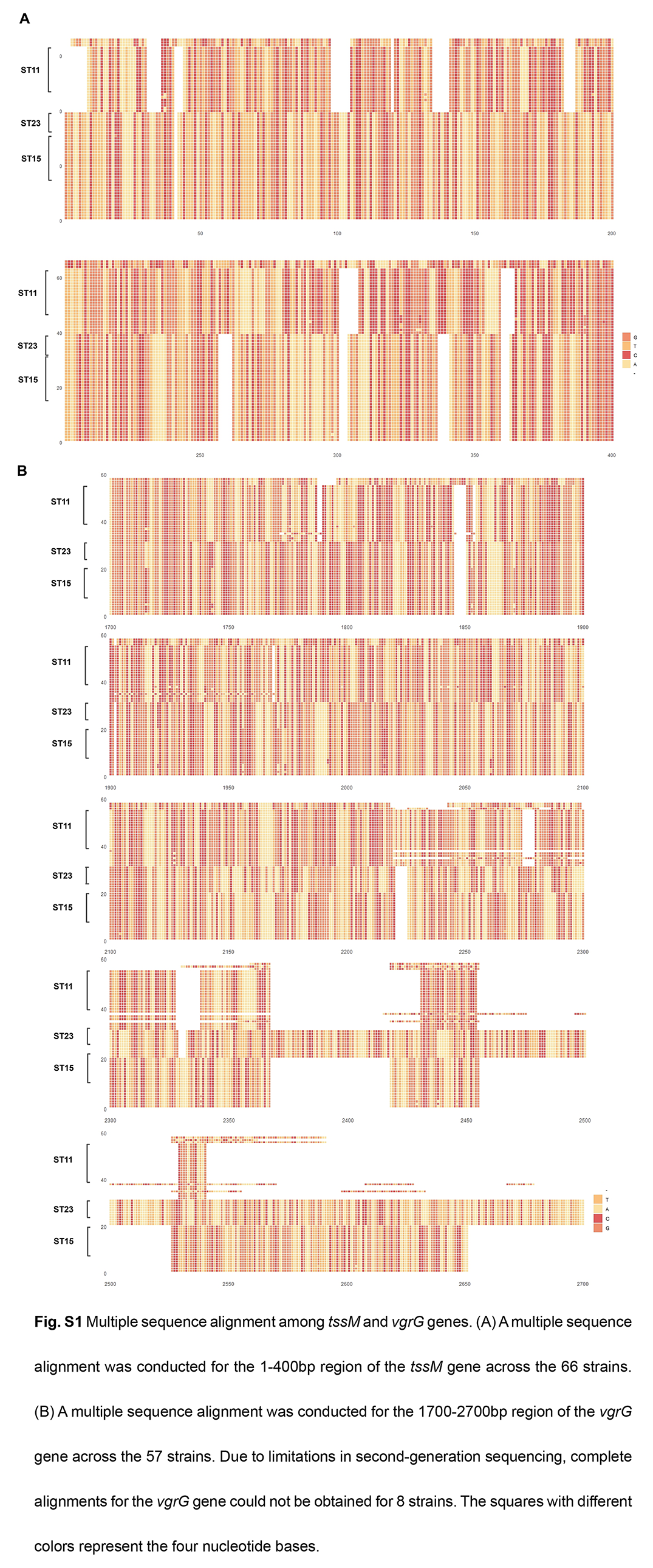

Supplement: Fig. S1 — Multiple sequence alignment among tssM and vgrG genes. [file msphere.00822-23-s0001.tiff]

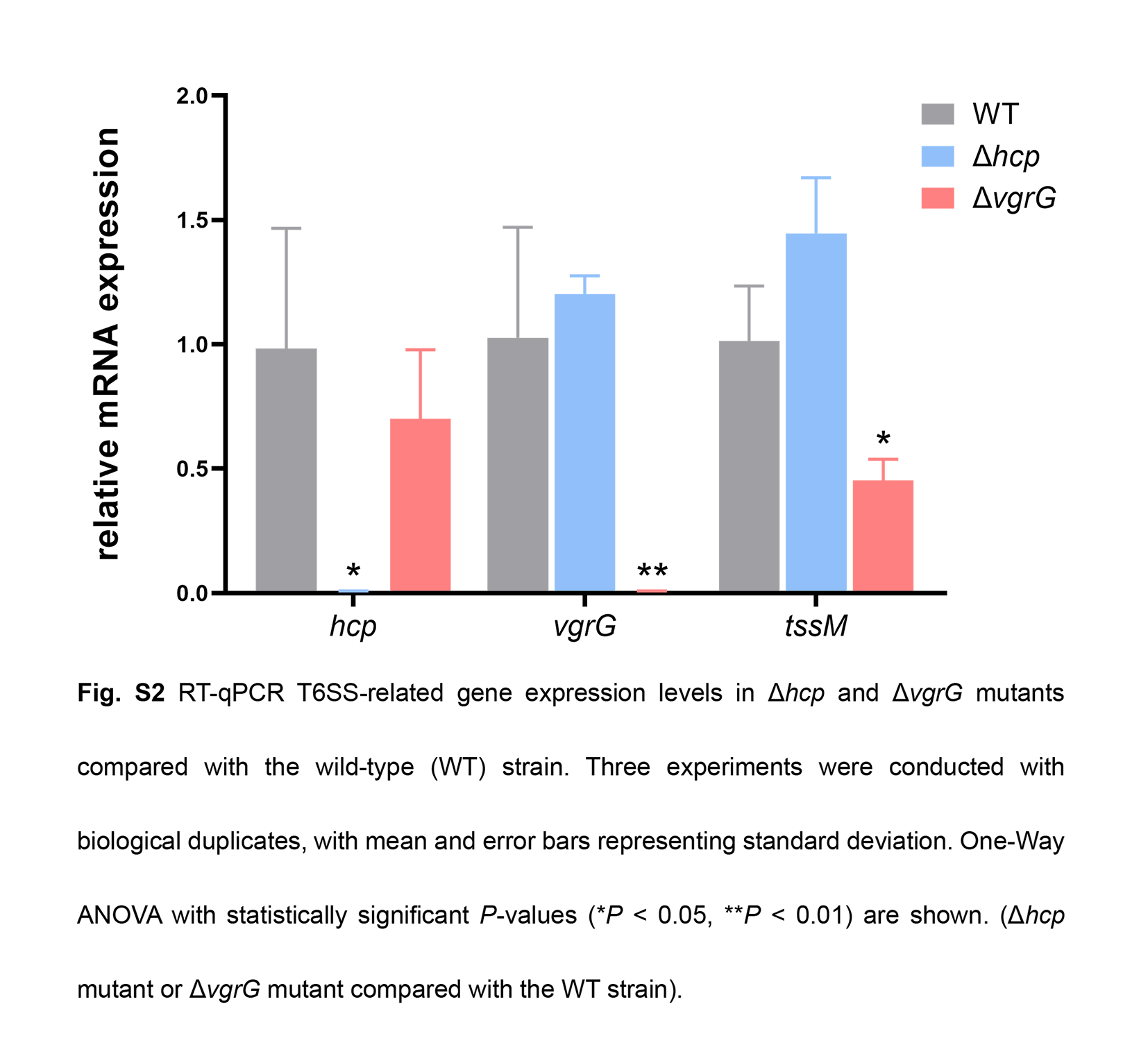

Supplement: Fig. S2 — RT-qPCR T6SS-related gene expression levels in Δhcp and ΔvgrG mutants compared with the wild-type strain. [file msphere.00822-23-s0002.tiff]

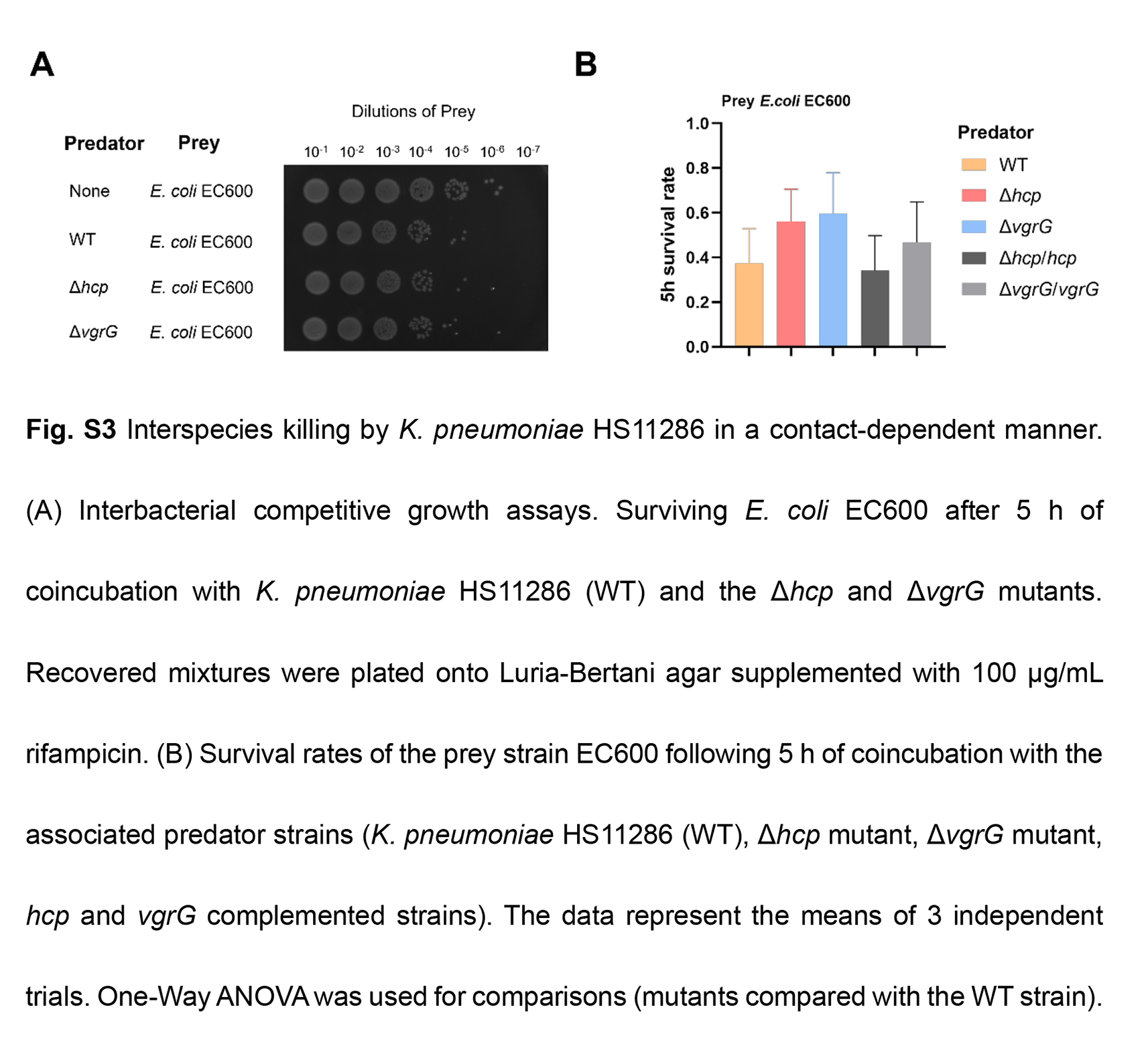

Supplement: Fig. S3 — Interspecies killing by K. pneumoniae HS11286 in a contact-dependent manner. [file msphere.00822-23-s0003.tiff]
