# Supplementary material for: A combination of genomics and transcriptomics provides insights into the distribution and differential mRNA expression of type VI secretion system in clinical Klebsiella pneumoniae
Source: mSphere. 2024 Mar 4;9(3):e00822-23. doi: 10.1128/msphere.00822-23 (PMC10964426; doi:10.1128/msphere.00822-23)
Supplement: Supplemental tables — Tables S1 to S5. [file msphere.00822-23-s0004.docx]

**Supplemental Material**

**TABLE S1.** Background information of 65 clinical *K. pneumoniae* strains used in this study.

| **Strains** | **biosample**  **accession** | **Collection date** | **Country** | **Host disease^a^** | **genome sizes** | **GC contents** | **Q20(%)** | **Q30(%)** | **Level** |
| --- | --- | --- | --- | --- | --- | --- | --- | --- | --- |
| KP172 | SAMN33040047 | 2017 | China | BSI | 5769163 | 0.5673 | 98.25 | 94.07 | draft |
| KP173 | SAMN33040048 | 2017 | China | HAP | 5798232 | 0.5664 | 98.03 | 93.39 | draft |
| KP175 | SAMN33040049 | 2017 | China | BSI | 5415067 | 0.5722 | 97.91 | 93 | draft |
| KP177 | SAMN33040050 | 2017 | China | BSI | 5942284 | 0.568 | 98.5 | 94.84 | draft |
| KP178 | SAMN33040051 | 2017 | China | BSI | 5346231 | 0.5736 | 98.09 | 93.56 | draft |
| KP179 | SAMN33040052 | 2017 | China | BSI | 5795591 | 0.5728 | 98.14 | 93.72 | draft |
| KP1714 | SAMN33040053 | 2017 | China | BSI | 5622017 | 0.5703 | 98.3 | 94.23 | draft |
| KP1716 | SAMN33040054 | 2017 | China | BSI | 5728936 | 0.5687 | 97.4 | 91.73 | draft |
| KP1727 | SAMN33040055 | 2017 | China | HAP | 5458498 | 0.5701 | 98.1 | 93.66 | draft |
| KP1728 | SAMN33040056 | 2017 | China | BSI | 5592475 | 0.5711 | 98.1 | 93.66 | draft |
| KP1729 | SAMN33040057 | 2017 | China | BSI | 5589931 | 0.5687 | 98.45 | 94.72 | draft |
| KP1736 | SAMN33040058 | 2017 | China | HAP | 5584146 | 0.5688 | 98.27 | 94.17 | draft |
| KP1746 | SAMN33040059 | 2017 | China | BSI | 5558939 | 0.5688 | 98.57 | 95.12 | draft |
| KP1750 | SAMN33040060 | 2017 | China | BSI | 5399808 | 0.5716 | 98.23 | 94.02 | draft |
| KP1751 | SAMN33040061 | 2017 | China | BSI | 5544439 | 0.5693 | 98.26 | 94.18 | draft |
| KP1756 | SAMN33040062 | 2017 | China | BSI | 5660095 | 0.5714 | 98.33 | 94.32 | draft |
| KP1759 | SAMN33040063 | 2017 | China | BSI | 5446131 | 0.5713 | 98.36 | 94.44 | draft |
| KP1760 | SAMN33040064 | 2017 | China | BSI | 5212122 | 0.5761 | 98.41 | 94.56 | draft |
| KP1764 | SAMN33040065 | 2017 | China | BSI | 5464967 | 0.5707 | 98.3 | 94.26 | draft |
| KP1765 | SAMN33040066 | 2017 | China | BSI | 5492128 | 0.5704 | 98.51 | 94.93 | draft |
| KP1766 | SAMN33040067 | 2017 | China | BSI | 5429979 | 0.5731 | 98.15 | 93.81 | draft |
| KP1767 | SAMN33040068 | 2017 | China | BSI | 5426398 | 0.5698 | 98.19 | 93.87 | draft |
| KP1769 | SAMN33040069 | 2017 | China | BSI | 5626339 | 0.5695 | 98.2 | 93.92 | draft |
| KP1874 | SAMN33040070 | 2018 | China | BSI | 5328923 | 0.5716 | 98.4 | 94.58 | draft |
| KP1875 | SAMN33040071 | 2018 | China | HAP | 5595322 | 0.5683 | 98.59 | 95.16 | draft |
| KP1880 | SAMN33040072 | 2018 | China | BSI | 5477557 | 0.5723 | 98.22 | 93.99 | draft |
| KP1881 | SAMN33040073 | 2018 | China | BSI | 5251465 | 0.573 | 98.43 | 94.66 | draft |
| KP1884 | SAMN33040074 | 2018 | China | BSI | 5655631 | 0.5698 | 98.41 | 94.59 | draft |
| KP1887 | SAMN33040075 | 2018 | China | BSI | 5625847 | 0.5704 | 98.29 | 94.22 | draft |
| KP1888 | SAMN33040076 | 2018 | China | BSI | 5535198 | 0.5691 | 98.38 | 94.52 | draft |
| KP1889 | SAMN33040077 | 2018 | China | BSI | 5533986 | 0.5692 | 98.71 | 95.58 | draft |
| KP1895 | SAMN33040078 | 2018 | China | HAP | 5674954 | 0.5677 | 98.27 | 94.14 | draft |
| KP1898 | SAMN33040079 | 2018 | China | HAP | 5584866 | 0.5692 | 97.88 | 92.87 | draft |
| KP18114 | SAMN33040080 | 2018 | China | BSI | 5823966 | 0.5642 | 98.54 | 95.05 | draft |
| KP18117 | SAMN33040081 | 2018 | China | BSI | 5413384 | 0.5697 | 98.33 | 94.36 | draft |
| KP193 | SAMN33040082 | 2019 | China | HAP | 5650989 | 0.5701 | 98.23 | 93.99 | draft |
| KP199 | SAMN33040083 | 2019 | China | HAP | 5781415 | 0.57 | 98.2 | 93.9 | draft |
| KP1912 | SAMN33040084 | 2019 | China | BSI | 5818540 | 0.5669 | 97.9 | 92.94 | draft |
| KP1913 | SAMN33040085 | 2019 | China | CNSI | 5609785 | 0.5705 | 97.96 | 93.14 | draft |
| KP1914 | SAMN33040086 | 2019 | China | BSI | 5602890 | 0.5705 | 98.25 | 94.06 | draft |
| KP1920 | SAMN33040087 | 2019 | China | BSI | 5816881 | 0.5671 | 98.19 | 94 | draft |
| KP1932 | SAMN33040088 | 2019 | China | BSI | 5616145 | 0.5686 | 98.41 | 94.6 | draft |
| KP1939 | SAMN33040089 | 2019 | China | BSI | 5582220 | 0.5687 | 98.09 | 93.85 | draft |
| KP1943 | SAMN33040090 | 2019 | China | BSI | 5332309 | 0.5738 | 97.86 | 93.02 | draft |
| KP1948 | SAMN33040091 | 2019 | China | HAP | 6024272 | 0.5612 | 98.03 | 93.32 | draft |
| KP1949 | SAMN33040092 | 2019 | China | LA | 5469500 | 0.5719 | 98.17 | 93.76 | draft |
| KP201 | SAMN33040093 | 2020 | China | BSI | 5827082 | 0.5662 | 98.31 | 94.26 | draft |
| KP203 | SAMN33040094 | 2020 | China | BSI | 5270238 | 0.5734 | 98.06 | 93.42 | draft |
| KP204 | SAMN33040095 | 2020 | China | BSI | 5822533 | 0.5665 | 98.2 | 93.9 | draft |
| KP207 | SAMN33040096 | 2020 | China | BSI | 5825635 | 0.5662 | 98.15 | 93.66 | draft |
| KP2011 | SAMN33040097 | 2020 | China | BSI | 5383163 | 0.5714 | 98.22 | 93.95 | draft |
| KP2019 | SAMN33040098 | 2020 | China | BSI | 5996605 | 0.5632 | 98.3 | 94.23 | draft |
| KP2021 | SAMN33040099 | 2020 | China | BSI | 5987755 | 0.5639 | 98.62 | 95.29 | draft |
| KP2022 | SAMN33040100 | 2020 | China | BSI | 5811233 | 0.5658 | 98.11 | 93.59 | draft |
| KP2027 | SAMN33040101 | 2020 | China | BSI | 5847066 | 0.5669 | 97.87 | 92.86 | draft |
| KP2030 | SAMN33040102 | 2020 | China | BSI | 5475150 | 0.5687 | 98 | 93.25 | draft |
| KP2031 | SAMN33040103 | 2020 | China | BSI | 5574514 | 0.5674 | 98.35 | 94.44 | draft |
| KP2032 | SAMN33040104 | 2020 | China | BSI | 5608469 | 0.5685 | 98.39 | 94.5 | draft |
| KP2033 | SAMN33040105 | 2020 | China | BSI | 5829146 | 0.5662 | 98.31 | 94.2 | draft |
| KP2034 | SAMN33040106 | 2020 | China | HAP | 5574643 | 0.5685 | 98.6 | 95.19 | draft |
| KP2035 | SAMN33040107 | 2020 | China | BSI | 5600268 | 0.5709 | 98.17 | 93.83 | draft |
| KP2036 | SAMN33040108 | 2020 | China | BSI | 5622360 | 0.5679 | 98 | 93.27 | draft |
| KP2037 | SAMN33040109 | 2020 | China | IAI | 5355315 | 0.5719 | 98.45 | 94.73 | draft |
| KP2038 | SAMN33040110 | 2020 | China | BSI | 5799054 | 0.5659 | 98.48 | 94.84 | draft |
| KP2039 | SAMN33040111 | 2020 | China | BSI | 5851275 | 0.5663 | 98.24 | 94.02 | draft |

^a^BSI: bloodstream infection; IAI: intra-abdominal infection; HAP: hospital acquired pneumonia; CNSI: central nervous system infection; LA: liver abscess; UNK: Unknown.

**TABLE S2.** Prevalence of T6SS-related genes in *K. pneumoniae* strains

| **T6SS - related gene** | **Strains** |
| --- | --- |
| *hcp* | 65 of 65 |
| *vgrG* | 61 of 65 |
| *tssM* | 21 of 65 |
| *hcp*,*vgrG*,*tssM* | 21 of 65 (32%) |

TABLE S3. T6SS-related gene expression was changed in Δ*hcp* and Δ*vgrG* mutants compared to the wild-type (WT) strain.

| Locus tag | Gene | log2 (Fold change) a | |
| --- | --- | --- | --- |
|  |  | Δ*hcp*/WT | Δ*vgrG*/WT |
| KPHS_22970 | *tssB* | - | - |
| KPHS_22980 | *tssC* | -1.5 | -1.7 |
| KPHS_22990 | *tssK* | -3.3 | -2.8 |
| KPHS_23000 | *tssL* | -3.3 | -3.6 |
| KPHS_23010 | *tagL* | -4.9 | -3.4 |
| KPHS_23020 | *tssD (hcp)* | NA | - |
| KPHS_23030 | *tssH* | - | -3.0 |
| KPHS_23040 | *tssI (vgrG)* | - | NA |
| KPHS_23120 | *paar* | - | - |
| KPHS_23140 | *tssM* | - | -1.7 |
| KPHS_23170 | *tssF* | - | -2.2 |
| KPHS_23180 | *tssG* | - | -2.7 |
| KPHS_23190 | *tssJ* | - | -4.2 |

^a^ NA，not applicable; -, no change.

**TABLE S4.** Strains and plasmids used in this study.

| **Strains/Plasmids** | **Features** | **Source** |
| --- | --- | --- |
| ***Klebsiella pneumoniae*** | |  |
| HS11286 (WT) | Clinical multidrug-resistant isolate | Laboratory |
| HS11286/ pHSG398 | HS11286 containing the empty vector pHSG398-Apr | This study |
| Δ*hcp* | HS11286 with a deletion of *hcp* gene | This study |
| Δ*hcp*/ pHSG398 | Δ*hcp* containing the empty vector pHSG398-Apr | This study |
| Δ*hcp*/ *hcp* | Δ*hcp* mutant carrying the pHSG398-Apr-*hcp* plasmid | This study |
| Δ*vgrG* | HS11286 with a deletion of *vgrG* gene | This study |
| Δ*vgrG*/ pHSG398 | Δ*vgrG* containing the empty vector pHSG398-Apr | This study |
| Δ*vgrG*/*vgrG* | Δ*vgrG* mutant carrying the pHSG398-Apr-*vgrG* plasmid | This study |
| ***Escherichia coli*** |  |  |
| DH5α | clone | TianGen, China |
| EC600 | rifampin-resistant | Laboratory |
| **Plasmid** |  |  |
| pKOBEG | Thermo-sensitive replicon (growth at 30°C); plasmid encode recombinant proteins for λ-Red homologous recombination; Apr^R^ | Laboratory |
| pMD18-T-Hyg | pMD18-T derived plasmid, plasmid for λ-Red homologous recombination; Hyg^R^ | Laboratory |
| pHSG398-Apr | pHSG398 derived plasmid, Apr^R^ | Laboratory |
| pHSG398-Apr-*hcp* | pHSG398-Apr containing the *hcp* gene from HS11286 | This study |
| pHSG398-Apr-*vgrG* | pHSG398-Apr containing the *vgrG* gene from HS11286 | This study |

**TABLE S5.** Primers used in this study.

| **Primer Name** | **Sequence (5’-3’)** |
| --- | --- |
| **For PCR** | |
| *hcp*-F | TCAGGATCGTGAAGGCAGTA |
| *hcp*-R | GCTATCGGAGTGAATGATGTTG |
| *vgrG*-F | GGCATCAATGTTCAGCAACC |
| *vgrG*-R | CACGCCATCAGCAAACTCC |
| *tssM*-F | CTGTGGCGGCTACGGTTAT |
| *tssM*-R | GATCTACGGTACGAGGCTCTG |
| **For the knockout of *hcp*** | |
| Δ*hcp*-KpnI-F | TACGAATTCGAGCTCGGTACCGCCGTTGCTGGAGGCGAT |
| Δ*hcp*-BamHI-R | GACGATATCTCTAGAGGATCCGGAATTACTCTCCATTGTGTAGTGAAG |
| Δ*hcp*-PstI-F | CGCTGCAGTTGCCCGTCATATTTCGC |
| Δ*hcp*-HindIII-R | CCAAGCTTAGCGCGGCTGTTTATCC |
| **For the knockout of *vgrG*** | |
| Δ*vgrG*-KpnI-F | TACGAATTCGAGCTCGGTACCAATCGACTTTCGCAACACCG |
| Δ*vgrG*-BamHI-R | GACGATATCTCTAGAGGATCCGCTACTCAT CGGATATTGTCCCC |
| Δ*vgrG*-PstI-F | AGAGATATCGTCGACCTGCAGATGACCAG ACAATGGATTGAGGA |
| Δ*vgrG*-HindIII-R | ACGACGGCCAGTGCCAAGCTTCCCTTGAT GCCTTTCGCC |
| **For construction of complement plasmid pHSG398-apr with *hcp* gene** | |
| *hcp*-KpnI-F | TACGAATTCGAGCTCGGTACCATGGCAAT TCCTGTTTATCTTTGG |
| *hcp*-PstI-R | GCTAAGCTTGCATGCCTGCAGTTACGCGG TGGCGCGTTC |
| **For construction of complement plasmid pHSG398-apr with *vgrG* gene** | |
| *vgrG*-KpnI-F | TACGAATTCGAGCTCGGTACCGTGAAATC GTTGTTGTTCAG |
| *vgrG*-PstI-R | AGCTAAGCTTGCATGCCTGCAGCTATGCC TCCCGCGGCACGA |
| **For qPCR** |  |
| q*hcp*-F | ATCCGCTGAGTTCAAGTG |
| q*hcp*-R | GGTTGTGCTTCTCGTAGG |
| q*vgrG*-F | TCGTTGTTGTTCAGCCATA |
| q*vgrG*-R | CCTTCATCAGCATCATCTCT |
| q*tssM*-F | AGTCAGATTGCTTCGGTAG |
| q*tssM*-R | CGGCGTCTAATAGTTGCT |
